# Effect of vertebrobasilar dolichoectasia on endovascular therapy in acute posterior circulation infarction

**DOI:** 10.3389/fnhum.2022.946349

**Published:** 2022-09-16

**Authors:** Jing Zhou, Daizhou Peng, Dong Sun, Weipeng Dai, Ceng Long, Renliang Meng, Jing Wang, Zhizhong Yan, Tao Wang, Li Wang, Chengsong Yue, Linyu Li, Wenjie Zi, Lingling Wang, Xiaoming Wang, Youlin Wu, Guohui Jiang

**Affiliations:** ^1^Department of Neurology, Affiliated Hospital of North Sichuan Medical College, Nanchong, China; ^2^Institute of Neurological Diseases, North Sichuan Medical College, Nanchong, China; ^3^Department of Neurology, Qianxinan People’s Hospital, Wuhan, China; ^4^Department of Neurology, Zhongnan Hospital, Wuhan University, Wuhan, China; ^5^Department of Neurology, Jiangmen Central Hospital, Jiangmen, China; ^6^Department of Emergency, Xiangtan Central Hospital, Xiangtan, China; ^7^Department of Neurology Affiliated Hospital of Southwest Medical University, Luzhou, China; ^8^Department of Neurology, Shanxi Provincial People’s Hospital, Taiyuan, China; ^9^Department of Neurosurgery, The 904th Hospital of the People’s Liberation Army, Wuxi, China; ^10^Department of Neurology, Huainan First People’s Hospital, Huainan, China; ^11^Department of Neurology, The First Affiliated Hospital of University of Science and Technology of China, Hefei, China; ^12^Department of Neurology, Xinqiao Hospital and the Second Affiliated Hospital, Army Medical University (Third Military Medical University), Chongqing, China; ^13^Department of Neurology, Chongzhou People’s Hospital, Chongzhou, China

**Keywords:** vertebrobasilar dolichoectasia, endovascular therapy, posterior circulation infarction, acute ischemic stroke, clinical outcome

## Abstract

**Background and purpose:**

This study aimed to analyze the feasibility and safety of endovascular therapy (EVT) in patients with acute posterior circulation stroke and vertebrobasilar dolichoectasia (VBD).

**Materials and methods:**

BASILAR was a national prospective registry of consecutive patients with symptomatic and imaging-confirmed acute stroke in the posterior circulation within 24 h of symptom onset. We evaluated EVT feasibility and safety in patients with VBD. Primary outcomes included improvement in modified Rankin Scale scores (mRS) at 90 days and mortality within 90 days. The secondary outcome was the rate of favorable functional outcome, defined as mRS ≤ 3 (indicating independent ambulation) at 90 days. Safety outcomes included surgery-related complications and other serious adverse events.

**Results:**

A total of 534 cases were included: 159 with VBD and 375 controls. No significant difference in mRS at 90 days was found between groups, but patients with VBD had a higher baseline National Institutes of Health Stroke Scale (NIHSS) score [30 (19–33) vs. 25 (15–32)] and were older [65 (59–74) vs. 63 (55–72) year]. After propensity score matching, there were no significant differences in baseline NIHSS score between the two groups, and the efficacy and safety of EVT were similar between patients with or without VBD. Furthermore, the prognostic effect of puncture-to-recanalization time on the probability of mortality within 90 days in EVT-treated patients with VBD was significant {adjusted odds ratio, 1.008 [95% confidence interval (1.001–1.015)]}.

**Conclusion:**

Endovascular therapy is safe and feasible in patients with acute posterior circulation stroke and VBD. The puncture-to-recanalization time is important for predicting the prognosis of EVT-treated patients with VBD.

## Introduction

Vertebrobasilar dolichoectasia (VBD) is a rare disease characterized by elongation, dilation, and tortuosity of the vertebrobasilar artery. Its prevalence is inaccurate and variable, ranging from 0.05 to 18%, based on several different study populations (Samim et al., 2016). For instance, in stroke patients, it ranges from 2.6 to 17.1% (Wolters et al., 2013). Despite the low prevalence of VBD, several serious clinical syndromes are associated with it, including combined brainstem and cranial nerve syndrome, cervicomedullary junction compression, temporary or permanent motor impairment, cerebellar dysfunction, central sleep apnea, hydrocephalus, ischemic stroke, and subarachnoid hemorrhage (Moss and West, 1996; Hongo et al., 1999). Despite various reports of combined compression of brainstem structures in patients with VBD, few studies focused on ischemic stroke complications and whether the treatment of vascular events associated with VBD must differ from standard treatment.

Recently, endovascular therapy (EVT) has been applied for its efficacy and safety. Several studies have shown better functional outcomes in patients with acute basilar artery occlusion (BAO) treated with EVT than patients treated with standard medical therapy alone and that EVT is effective in reducing mortality and improving activities of daily function in stroke patients (Kang et al., 2018; Kwak and Park, 2020). The BASILAR study found among patients with acute BAO, EVT administered within 24 h of estimated occlusion time is associated with better functional outcomes and reduced mortality (Writing Group for the et al., 2020). In other words, EVT is an effective treatment option for patients with ischemic stroke in the posterior circulation; however, the effect of EVT on patients with ischemic stroke and VBD is unclear.

Based on the largest multicenter consecutive BAO-cohort undergoing EVT, this study investigated for the first time whether EVT affects outcomes in patients with VBD and ischemic stroke in the posterior circulation.

## Materials and methods

Data supporting this study may be provided by the corresponding author upon reasonable request.

### The BASILAR registry and patient selection

The BASILAR study enrolled patients with symptomatic and radiologically confirmed acute stroke in the posterior circulation within 24 h of symptom onset from 47 comprehensive stroke centers in China between January 2014 and May 2019. Study centers were required to have performed at least 30 endovascular procedures annually, including at least 15 thrombectomy procedures using stent retriever devices. All interventionists had to be certified in EVT of large vessel occlusion strokes. The study protocol was approved by the ethics committee of the Xinqiao Hospital, Army Medical University, in Chongqing, China, and each subcenter. BASILAR is registered on the Chinese Clinical Trial Registry (Writing Group for the et al., 2020).

Currently, there are no uniform quantitative imaging criteria for VBD diagnosis. It usually relies on the practical experience of clinicians and radiologists, combined with the assessment of clinical symptoms on vascular images. Patients are diagnosed with VBD depending on the extrapolation of the established computed tomography (CT) criteria by Smoker (Smoker et al., 1986) and magnetic resonance imaging (MRI) criteria by Giang et al. (1988). According to the imaging diagnostic criteria, we included data of patients with VBD if they fulfilled the following criteria: (1) aged ≥ 18 years; (2) presentation within 24 h of estimated time of acute stroke in the posterior circulation; (3) being able to provide informed consent; (4) EVT had to be initiated within 24 h of estimated time of acute stroke in the posterior circulation; (5)diameter of the vertebral artery or BA > 4.5 mm; (6) for the basilar artery (BA), bifurcation above the suprasellar cistern or evidence of any portion lateral to the margin of the clivus or dorsum sellae considered elongated; (7) length of the BA > 29.5 mm or lateral deviation at 10 mm from the vertical line between the origin and bifurcation of the BA; and (8) if the length of the intracranial segment of the vertebral artery is > 23.5 mm or any vertebral artery deviates > 10 mm from the vertical line at the beginning of the BA, the vertebral artery will be lengthened. We included data of patients without VBD if they fulfilled the first four criteria. Exclusion criteria were: (1) lack of follow-up information, (2) incomplete baseline critical imaging data, (3) patients with occlusion in the BA on angiography, (4) neuroimaging evidence of cerebral hemorrhage on presentation, (5) current pregnancy or lactation, and (6) a serious, advanced, or terminal illness.

All participating centers’ ethics committees approved the study protocol. Written informed consent was obtained from all patients or their legal representatives.

### Variables and imaging analysis

Patients were divided into two groups based on initial enrollment criteria: the VBD and non-VBD groups. Stroke severity was assessed at admission using the National Institutes of Health Stroke Scale (NIHSS) score. Imaging scans were separately reviewed by two trained neuroradiologists (Dr. Chen and Dr. Qiu). In case of discrepancies in the assessment, a third neurologist (Dr. Zi) made the final decision, and a final retrospective analysis was performed. Vascular risk factors (diabetes, hypertension, smoking, hypercholesterolemia, and coronary artery disease) were compared with those in non-VBD patients.

### Outcome measurement

Modified Rankin Scale scores (mRS) is a 7-point scale for neurological disability [ranging from 0 (asymptomatic) to 6 (death)] and is assessed by a local neurologist.

The secondary clinical endpoints included functional independence mRS ≤ 3 at 90 days, while other outcomes included changes in the NIHSS score from baseline at 24 h and at 5–7 days (or discharge, if earlier).

Safety outcomes included symptomatic intracranial hemorrhage (sICH) within 48 h, confirmed by CT or MRI, and procedure-related complications or other serious adverse events. sICH was defined as an NIHSS score ≥ 4 or any parenchymal intracerebral hemorrhage.

### Statistical analysis

We compared baseline characteristics, outcomes, and severe adverse events between the VBD and non-VBD groups. Statistical analyses were performed using SPSS version 26 (IBM Corp., Armonk, NY, United States) and STATA version 15.2 (Stata Corp LLC, TX, United States). A two-tailed *P* < 0.05 was considered statistically significant. Univariate comparisons were performed using Fisher’s exact test or χ^2^ test for categorical variables and Kruskal Wallis test or Mann–Whitney U test for continuous variables. VBD cases were matched in a 1:1 ratio to controls based on the propensity score. To assess the prognostic impact of EVT, propensity score matching was applied to achieve a balance at baseline. We performed a 1:1 matching based on the nearest-neighbor matching algorithm using a caliper width of 0.2 of the propensity score, with age and baseline NIHSS as covariates (Methods in Supplementary material).

The second part of the analysis included only patients in the VBD group. A binary logistic regression model was used to assess the variables independently associated with 90-days favorable functional outcomes. The association of puncture-to-recanalization time with outcome is shown in the adjusted margin plots.

## Results

### Patients’ characteristics

Based on the inclusion and exclusion criteria, we initially screened 829 patients from 47 comprehensive stroke centers in China. Among these patients, 179 patients were excluded because the data couldn’t be measured due to basilar artery remained closed after surgery and 116 patients were excluded because of lack of imaging data, leaving 534 patients as the current study population.

### Baseline characteristics

A total of 534 cases were reviewed, revealing 159 patients with VBD and 375 patients without VBD who met the inclusion criteria. Table 1 shows the baseline patients’ characteristics. VBD was more common in men (81.8%). Hypertension was present in 103 cases (64.8%) and diabetes mellitus in 31 patients (19.5%). Hyperlipidemia and coronary heart disease were present in 47 (29.6%) and 20 (12.6%) patients, respectively. Compared with the non-VBD group, patients in the VBD group were older [65 (59–74) vs. 63 (55–72)] and had higher baseline NIHSS scores [30 (19–33) vs. 25 (15–32)]. Other baseline characteristics were not significantly different between the two groups.

**TABLE 1:** Baseline characteristics of patients with or without VBD.

	No./Total No. (%)			Propensity Score Matching No./No. (%)		
Characteristic	Non-VBD, *n* = 375	VBD, *n* = 159	*P*-value	Non-VBD, *n* = 159	VBD, *n* = 159	*P*-value
Age, median (IQR), y	63 (55–72)	65 (59–74)	< 0.015	64 (57–74)	65 (59–74)	0.306
Male, *n*/total *n* (%)	277 (73.9)	130 (81.8)	0.05	121 (76.1)	130 (81.8)	0.216
Baseline NIHSS, median (IQR)	25 (15–32)	30 (19–33)	0.03	29 (20–34)	30 (19–34)	0.793
pc-ASPECT baseline	8 (7–9)	8 (7–9)	0.7	8 (7–9)	8 (7–9)	0.627
ASITN/SIR grade			0.23			0.478
Grade 0–1	220 (58.7)	100 (62.9)		100 (62.9)	100 (62.9)	
Grade 2	99 (26.4)	44 (27.7)		38 (23.9)	44 (27.7)	
Grade 3–4	56 (14.9)	15 (9.4)		21 (13.2)	15 (9.4)	
Medical history *n*/total *n* (%)						
Ischemic stroke	85 (22.7)	35 (22.0)	0.868	39 (24.5)	35 (22.0)	0.596
Hypertension	263 (70.1)	103 (64.8)	0.223	119 (74.8)	103 (64.8)	0.051
Hyperlipidemia	132 (35.2)	47 (29.6)	0.207	50 (31.4)	47 (29.6)	0.715
Diabetes	89 (23.7)	31 (19.5)	0.283	42 (26.4)	31 (19.5)	0.142
Smoking	147 (39.2)	52 (32.7)	0.156	70 (44)	52 (32.7)	0.038
Dringking	95 (25.3)	31 (19.5)	0.146	43 (27)	31 (19.5)	0.111
Coronary heart disease	54 (14.4)	20 (12.6)	0.577	28 (17.6)	20 (12.6)	0.21
Atrial fibrillation	76 (20.3)	29 (18.2)	0.590	38 (23.9)	29 (18.2)	0.216
Location of occlusion, *n*/total *n* (%)			0.732			0.4
Distal BA	115 (30.7)	45 (28.3)		50 (31.4)	45 (28.3)	
Middle BA	113 (30.1)	53 (33.3)		45 (28.3)	53 (33.3)	
Proximal BA	64 (17.1)	32 (20.1)		24 (44.8)	32 (20.1)	
VA-V4	73 (19.5)	25 (15.7)		35 (58.3)	25 (15.7)	
**Time metrics, min, median (IQR)**						
Onset-puncture time	315 (216–459)	355 (208–510)	0.184	319 (219–490)	355 (208–510)	0.486
Onset-imaging time	190 (80–332)	232 (102–410)	0.048	220 (100–389)	232 (102–410)	0.802
Puncture-recanalization time	100 (69–141)	110 (77–160)	0.032	102 (67–141)	110 (77–160)	0.032
mTICI score 2b/3, *n*/total *n* (%)	303 (80.8)	131 (82.4)	0.667	125 (78.6)	131 (82.4)	0.396

IQR, interquartile range; VBD, vertebrobasilar dolichoectasia; Non-VBD, patients without vertebrobasilar dolichoectasia; NIHSS, National Institutes of Health Stroke Scale; pc-ASPECTS, posterior circulation Alberta Stroke Program Early Computed Tomography Score; VA-V4, V4 of vertebral artery; mTICI, modified Thrombolysis in Cerebral Infarction Score. ASITN/SIR grade indicates the American Society of Interventional and Therapeutic Neuroradiology/Society of Interventional Radiology collateral score.

### Primary efficacy outcome

**FIGURE 1:**
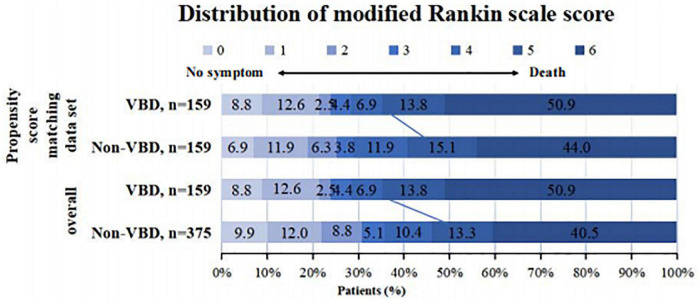
Distribution of the modified Rankin scale score at 90 days in the VBD and Non-VBD.

The Distribution of the modified Rankin scale score at 90 days is shown in Figure 1. The analysis of the primary outcome is shown in Table 2. The median 90-day mRS score was 6 [interquartile range (IQR) (3–6)] in the VBD group and 5 [IQR (2–6)] in the non-VBD group (*P* < 0.05; Table 2). However, after adjusting for confounding factors, there was no statistical difference between groups in mRS at 90 days. Mortality at 90 days was significantly higher in the VBD group than in the non-VBD group [81 of 159 patients (50.9%) vs. 152 of 357 patients (40.5%)]. Similarly, there was no significant difference between the two groups after adjusting for confounding factors.

**TABLE 2:** Primary and secondary efficacy outcomes and safety outcomes.

	No./Total No. (%)							Propensity score matching No./No. (%)		
		
Characteristic	Non-VBD *n* = 375	VBD *n* = 159	*P*-value	Unadjusted odds ratio (95% CI)	*P*-value	Adjused odds^a^ ratio (95% CI)	*P*-value^b^	Non-VBD *n* = 159	VBD *n* = 159	*P*-value
Primary outcome										
mRS score at 90 days (ordinal)	5 (2–6)	6 (3–6)	0.042	0.696 (0.493–0.983)	0.04	0.9 (0.626–1.293)	0.569	5 (3–6)	6 (3–6)	0.689
Mortality at 90 days	152 (40.5)	81 (50.9)	0.027	1.542 (1.049–2.212)	0.027	1.162 (0.768–1.759)	0.477	74 (46.5)	81 (50.9)	0.432
Secondary outcome										
mRS 0-3 at 90 days	134 (35.7%)	45 (28.3)	0.096					49 (30.8)	45 (28.3)	0.623
mRS 0-2 at 90 days	115 (30.7)	38 (23.9)	0.114					38 (23.9)	38 (23.9)	1
mTICI			0.667							0.369
0–2a	72 (19.2)	28 (17.6)						33 (21.4)	28 (17.6)	
2b–3	303 (80.8)	131 (82.4)						125 (78.6)	131 (82.4)	
NIHSS score, median (IQR)										
NIHSS score after 24 h	26 (11–35)	30 (16–35)	0.036	1.176 (1.144–1.210)	0.001	1.179 (1.145–1.215)	0.001	30 (17–36)	30 (16–35)	0.483
NIHSS score at 5–7 days	18 (5.5–35)	25 (7.3–35)	0.074					24 (8–36)	25 (7.3–35)	0.713
Safety outcome										
sICH	24 (6.5)	14 (9.0)	0.512					12 (7.6)	14 (9)	0.88
Respiratory Failure	151 (40.3)	66 (41.5)	0.789					73 (45.9)	66 (41.5)	0.429
Reocclusion	12 (8.7)	6 (15)	0.244					5 (9.8)	6 (15)	0.45
Venous Thrombosis	31 (8.3)	7 (4.4)	0.112					12 (7.5)	7 (4.4)	0.237

^a^The multiple logistic regression test was used to analyze odds ratios. Adjusted variables: age, baseline NIHSS score, and time from puncture to recanalization.

^b^The Bonferroni correction method was applied to multiple comparisons using a *P*-value < 0.05/number of comparisons as a threshold for statistical significance.

CI, confidence interval; mRS, modified Rankin Scale; sICH, symptomatic intracranial hemorrhage; mTICI, modified Thrombolysis in Cerebral Infarction Score; NIHSS, National Institutes of Health Stroke Scale; IQR, interquartile range; VBD, vertebrobasilar dolichoectasia; Non-VBD, patients without vertebrobasilar dolichoectasia.

### Secondary efficacy outcomes

The secondary outcomes are shown in Table 2. There was no significant difference in the proportion of favorable prognosis (mRS ≤ 3) at 90 days between the two groups; the baseline NIHSS score at 24 h was significantly higher in the VBD group than in the non-VBD group [30 (16–35) vs. 26 (11–35); *P* < 0.05], with an adjusted odds ratio (OR) of 1.179 [95% confidence interval (CI) (1.145–1.215), *P* < 0.05], there was no significant difference between the two groups after adjusting for confounding factors.

### Propensity score matching analysis

Table 1 shows that baseline characteristics were well balanced between the two groups after 1:1 propensity score matching. The median 90–day mRS was 6 [IQR (3–6)] in the VBD group and 5 [IQR (3–6)] in the non-VBD group (*P* = 0.689); there was no significant difference between the two groups, and mortality within 90 days occurred in 81 of 159 patients (50.9%) in the VBD group and 74 of 159 patients (46.5%) in the non-VBD group [81 (50.9%) vs. 74 (46.5%), *P* = 0.432]. The rate of sICH was 9% (14 of 159 patients) in the VBD group and 7.6% in the non-VBD group (12 of 159 patients; *P* = 0.429). There was no significant difference between surgery-related complications and other severe adverse events.

### Outcomes of vertebrobasilar dolichoectasia with endovascular therapy

Restricting the analysis to only the VBD group, the results of univariate and logistic regression analyses are presented in Tables 3, 4. Patients were divided into favorable and unfavorable outcome groups based on prognosis (mRS ≤ 3) at 90 days.

**TABLE 3:** Factors associated with favorable outcomes at 90 days of VBD patients.

	Favorable outcome (*n* = 45)	Unfavorable outcome (*n* = 114)	*P*-value	Adjusted OR (95% CI)*^a^*	*P*-value^b^
Age, years, median (IQR)	63 (54.5–70.5)	67 (60.8–75.3)	0.24	0.948 (0.910–0.989)	0.013
Male, no./total no. (%)	35 (77.8)	95 (83.3)	0.414	0.519 (0.173–1.554)	0.241
Baseline NIHSS, median (IQR)	21 (9–30)	30.5 (24–35)	< 0.001	0.938 (0.893–0.985)	0.01
Baseline ASPECTS, media (IQR)	9 (8–10)	7 (6–8)	< 0.001	1.839 (1.325–2.553)	< 0.001
**History, no./total no. (%)**					
Diabetes	5 (11.1)	26 (22.8)	0.094	0.512 (0.143–1.839)	0.305
Coronary heart disease	5 (11.1)	15 (13.2)	0.726	0.618 (0.160–2.380)	0.484
Atrial fibrillation	9 (20)	20 (17.5)	0.718	1.178 (0.387–3.591)	0.773
Location of occlusion, no./total no. (%)			0.139		
Distal BA	19 (42.2)	30 (26.3)		Reference	Reference
Middle BA	12 (26.7)	41 (36)		0.180 (0.055–0.592)	0.005
Proximal BA	10 (22.2)	22 (19.3)		0.586 (0.173–1.987)	0.391
VA-V4	4 (8.9)	21 (18.4)		0.117 (0.020–0.689)	0.018
Intravenous thrombolysis, no./total no. (%)	8 (17.8)	16 (14)	0.553	1.277 (0.410–3.974)	0.673
Onset-Imaging Time, min, median (IQR)	212 (87–406.5)	240 (104.3–417)	0.511	1.000 (0.998–1.002)	0.728
Puncture-Recanalization Time, min, median (IQR)	99 (66.5–133.5)	113 (86–167.5)	0.028	0.996 (0.988–1.004)	0.319
Collateral grade, *n* (%)			< 0.001		
ASTIN/SIR grade 0	5 (11.1)	38 (33.3)		Reference	Reference
ASTIN/SIR grade 1	11 (24.4)	46 (40.4)		1.181 (0.341–4.096)	0.793
ASTIN/SIR grade 2	18 (40)	26 (22.8)		1.938 (0.527–7.130)	0.319
ASTIN/SIR grade 3	11 (24.4)	4 (3.5)		5.538 (0.960–31.941)	0.056
ASTIN/SIR grade 4	NA	NA	NA	NA	NA

^a^The multiple logistic regression test was used to analyze ORs. Adjusted variables: baseline NIHSS score, Baseline ASPECTS, Puncture-Recanalization Time, Collateral grade.

^b^The Bonferroni correction method was applied to multiple comparisons using a *p*-value < 0.05/number of comparisons as a threshold for statistical significance.

NIHSS, National Institutes of Health Stroke Scale; ASITN/SIR grade indicates the American Society of Interventional and Therapeutic Neuroradiology/Society of Interventional Radiology collateral score; ASPECTS, Acute Stroke Prognosis Early Computed Tomography Score; IQR, interquartile range; CI, confidence interval.

**TABLE 4:** Factors associated with mortality at 90 days of VBD patients.

	Death (*n* = 81)	*P*-value	Adjusted OR (95% CI)^a^	*P*-value^b^
Age, years, median (IQR)	66 (59–75.5)	0.742	1.005 (0.971–1.040)	0.777
Male, no./total no. (%)	65 (80.2)	0.614	0.577 (0.212–1.567)	0.281
Baseline NIHSS, median (IQR)	32 (26.5–35)	< 0.001	1.121 (1.064–1.181)	< 0.001
Baseline ASPECTS, media (IQR)	7.5 (6–9)	0.008	0.804 (0.637–1.015)	0.066
**History, no./total no. (%)**				
Diabetes	20 (24.7)	0.092	2.358 (0.828–6.719)	0.108
Coronary heart disease	7 (8.6)	0.127	0.372 (0.121–1.142)	0.084
Atrial fibrillation	15 (18.5)	0.926	0.911 (0.351–2.367)	0.848
Location of occlusion, no./total no. (%)		0.775		
Distal BA	22 (27.2)		Reference	Reference
Middle BA	28 (34.6)		1.713 (0.651–4.506)	0.275
Proximal BA	17 (21)		1.753 (0.599–5.131)	0.306
VA-V4	14 (17.3)		2.144 (0.650–7.069)	0.21
Intravenous thrombolysis, no./total no. (%)	12 (14.8)	0.92	1.250 (0.451–3.463)	0.668
Onset-Imaging Time, min, median (IQR)	237 (103.5–398.2)	0.973	1.000 (0.998–1.001)	0.657
Puncture-Recanalization Time, min, median (IQR)	123 (95.5–171)	0.003	1.008 (1.001–1.015)	0.02
Collateral grade, *n* (%)		0.001		
ASTIN/SIR grade 0	26 (32.1)		Reference	Reference
ASTIN/SIR grade 1	35 (43.2)		1.749 (0.684–4.473)	0.243
ASTIN/SIR grade 2	19 (23.5)		1.755 (0.594–5.187)	0.309
ASTIN/SIR grade 3	1 (1.2)		0.189 (0.019–1.881)	0.155
ASTIN/SIR grade 4	NA	NA	NA	NA

^a^The multiple logistic regression test was used to analyze odds ratios. Adjusted variables: baseline NIHSS score, Baseline ASPECTS, Puncture-Recanalization Time, Collateral grade.

^b^The Bonferroni correction method was applied to multiple comparisons using a *P*-value < 0.05/number of comparisons as a threshold for statistical significance.

NIHSS, National Institutes of Health Stroke Scale; ASITN/SIR grade indicates the American Society of Interventional and Therapeutic Neuroradiology/Society of Interventional Radiology collateral score; IQR, interquartile range; CI, confidence interval.

**FIGURE 2:**
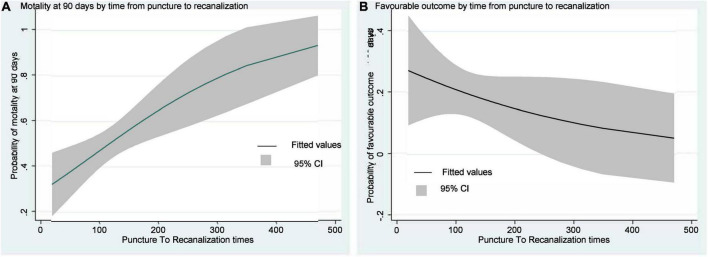
Curves show the increase in predicted motality probabilities with increases in puncture-to-recanalization time and **(A)**. The decreases in predicted favorable outcome probabilities with increases in puncture-to-recanalization time **(B)**.

**FIGURE 3:**
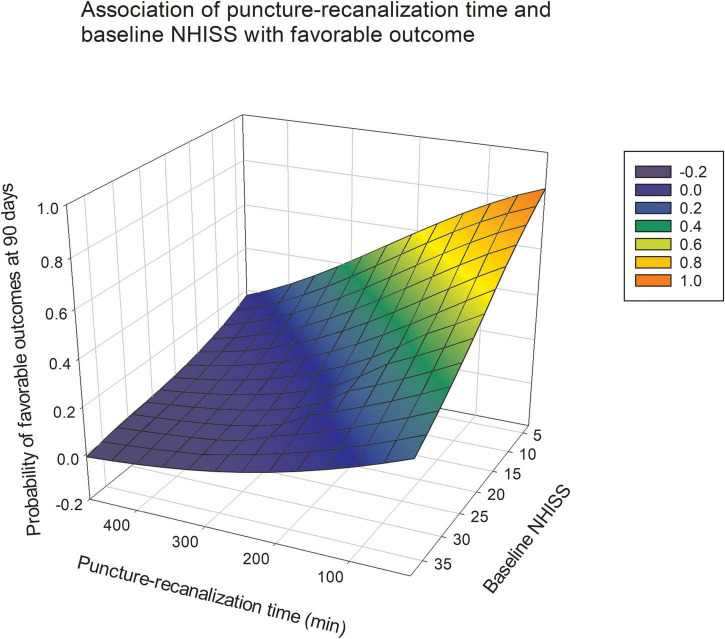
The gray shading indicates 95% CIs. Association of NIHSS and time from puncture to recanalization with the probability of a favorable outcome at 90 days after endovascular thrombectomy.

The odds of baseline NIHSS score significantly decreased by 9% in the favorable outcome group [adjusted OR 0.938, 95% CI (0.893–0.985)]. Similarly, the odds of baseline NIHSS score significantly increased by 11.2% in the probability of mortality within 90 days in the unfavorable outcome group [adjusted OR 1.121, 95% CI (1.064–1.181)]. We observed a significant detrimental effect of prolonging the puncture-to-recanalization time on the probability of mortality within 90 days [adjusted OR 1.008, 95% CI (1.001–1.015)] (Table 4). Figure 2 illustrates the predicted outcome probabilities with puncture-to-recanalization time increment as a continuous variable. Figure 3 illustrates the effects of baseline NIHSS score and puncture-to-recanalization time on the probability of favorable outcomes with EVT.

## Discussion

To our knowledge, this study is the first and largest prospective multicenter registry of patients with VBD. The main finding of the study was the correlation between EVT and the feasibility and safety in patients with acute posterior circulation stroke and VBD. And there was no statistical difference in the incidence of adverse events between the EVT and non-EVT group. The findings provide extensive evidence for the choice of EVT for patients with VBD and for clinicians.

Many studies have confirmed the efficacy and safety of EVT after intervention in patients with acute BAO (Gory et al., 2016; Kang et al., 2018; Kwak and Park, 2020). Mechanical thrombectomy for acute ischemic stroke caused by anterior or posterior circulation occlusion of large vessels is safe and effective in a recent randomized controlled trial (Berkhemer et al., 2015; Writing Group for the et al., 2020). To our knowledge, no other trials have evaluated the safety and efficacy of mechanical embolization in stroke patients with VBD. Our results clearly demonstrate the feasibility and safety of EVT in patients with VBD. Our study retrospectively analyzed the clinical and imaging data of 159 VBD patients with mechanical embolization in the acute phase of cerebral infarction to investigate the feasibility and safety of this technique in preventing ischemic events in patients with VBD. Our study showed that patients with VBD had a higher NIHSS score and probability of death at 90 days; after adjusting for confounding factors, there was no significant difference in mortality at 90 days between the two groups. This may be because EVT treatment can relieve symptoms in patients with acute posterior circulation infarction combined with VBD. In addition, we found no significant differences in any safety endpoints between groups. Our study did not find evidence of differences in neurological outcomes between VBD and non-VBD patients. Therefore, EVT may be acceptable for patients with acute cerebral infarction associated with VBD.

However, there are few reports on the puncture-to-recanalization time; therefore, we also paid attention to the effect of operation time on the prognosis of EVT. Recent studies reporting the effect of procedural time on EVT outcomes have shown that prolonged procedural time not only decreases functional independence but also increases the incidence of complications (Spiotta et al., 2014; Hassan et al., 2019). Furthermore, as our results and those of other studies have shown, patients with good outcomes typically had a much shorter puncture-to-recanalization time, supporting that a shorter puncture-to-recanalization time may reduce the risk of death. Therefore, finding a more appropriate method during surgery may also be a key factor affecting the prognosis of patients.

Vertebrobasilar dolichoectasia is a potentially serious disease that can lead to severe disability due to ischemic or compressive disease of the posterior circulation. Symptomatic VBD is generally considered to have a poor outcome and to be a challenging disease for which there are no effective treatment modalities. No effective treatment is available for some asymptomatic patients. Previous studies have found patients with VBD and hypertension, previous anterior or posterior stroke, and lack of warfarin therapy may have a higher risk of all-cause mortality. Patients with VBD and diabetes mellitus, smoking, and BA involvement may have a higher risk of posterior circulation dysfunction; (Wolfe et al., 2008) therefore, the prognosis of patients with VBD appears more dependent on traditional vascular risk factors. However, we were unable to find the correlation between these risk factors with prognostic factors. It may be that EVT was not involved in previous studies, but in our study, EVT intervention was performed after the onset of the patients with VBD. However, as this is a retrospective study, no additional information was available to assess the effectiveness of the treatment for VBD or onset of ischemia. As mentioned previously, our study confirmed the safety of EVT in patients with acute posterior circulation infarction associated with VBD, and further studies are required to develop safe and feasible treatments to prevent ischemic events in patients with VBD.

### Limitations

Our study has all the inherent limitations of a non-randomized study. The major ones were its retrospective nature, selection bias, and the self-assessed angiographic evaluation. Because we lacked data on the mechanism of stroke death, our study did not yet answer whether VBD is a direct cause of death or a marker of high risk of death from cerebrovascular disease. Additionally, the lack of properly validated diagnostic criteria for VBD is a potential limitation. Nevertheless, this is the largest study published to date on VBD This study could provide additional information on treatment strategies for this subgroup of cerebral infarction, and further prospective studies are required to evaluate the best treatment options for VBD.

## Conclusion

In conclusion, patients with VBD can benefit from EVT after acute cerebral infarction. In addition, prolonging procedural time may reduce the likelihood of a favorable functional outcome in patients with VBD treated with EVT. Strategies that reduce surgery duration may provide opportunities for improving patient prognosis.

## Data availability statement

The raw data supporting the conclusions of this article will be made available by the authors, without undue reservation.

## Ethics statement

The studies involving human participants were reviewed and approved by the Medical Ethics Committee of Second Affiliated Hospital of Third Military Medical University. The patients/participants provided their written informed consent to participate in this study.

## Author contributions

JZ and DP analyzed and interpreted the data and drafted the manuscript. DS, WD, CL, RM, JW, ZY, TW, LW, and CY assisted to promote data collection. LlW and GJ completed the statistical work. WZ, YW, and XW conceived and designed the research. All authors contributed to the article and approved the submitted version.
