# Supplementary material for: Effect of vertebrobasilar dolichoectasia on endovascular therapy in acute posterior circulation infarction
Source: Front Hum Neurosci. 2022 Sep 16;16:946349. doi: 10.3389/fnhum.2022.946349 (PMC9523604; doi:10.3389/fnhum.2022.946349)
Supplement: Supplementary file 1 [file Data_Sheet_1.PDF]

We performed a 1:1 propensity score matching based on the nearest-neighbor matching algorithm with a caliper width of 0.2 using SPSS 23.0 (IBM SPSS Statistics). The variables, parameter settings, and results are listed as follows:

Propensity Score Matching

Sample Sizes								
	All		Matched		Unmatched		Discarded	
Subsamples	Control	Treated	Control	Treated	Control	Treated	Control	Treated
(all cases)	375	159	159	159	216	0	0	0

**Overall balance test (Hansen & Bowers, 2010)**				
	chi-square	df	p-value	
(all cases)	.073	2.000	.964	

Relative multivariate imbalance L1 (Iacus, King, & Porro, 2010)

	Before matching	After matching
(all cases)	.349	.365

**Summary of unbalanced covariates (|d| > .25)**	No covariate exhibits a large imbalance (|d| > .25).

R 图

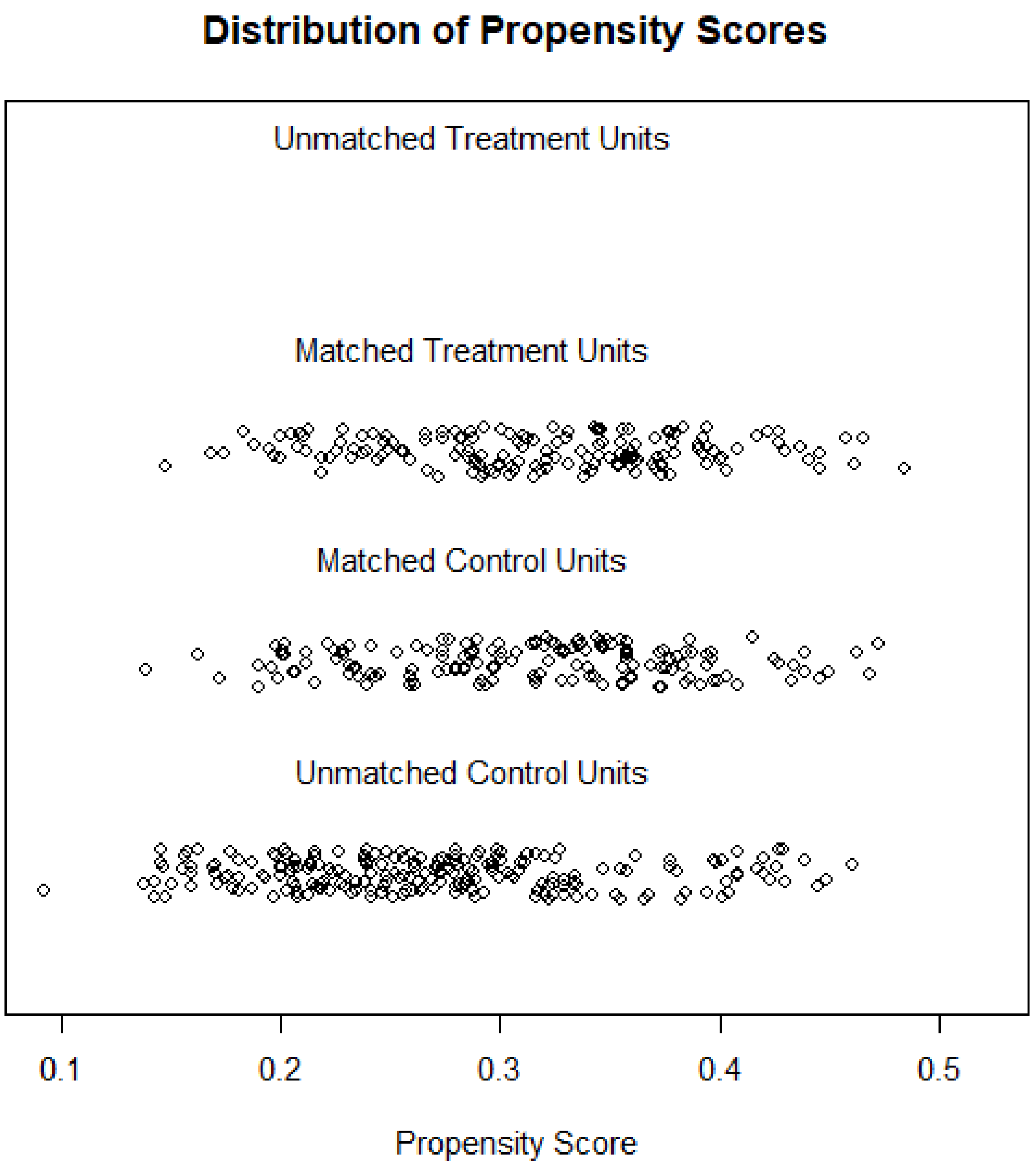

Distribution of Propensity Scores
Unmatched Treatment Units
Matched Treatment Units
Matched Control Units
Unmatched Control Units
0.1
0.2
0.3
0.4
0.5
Propensity Score

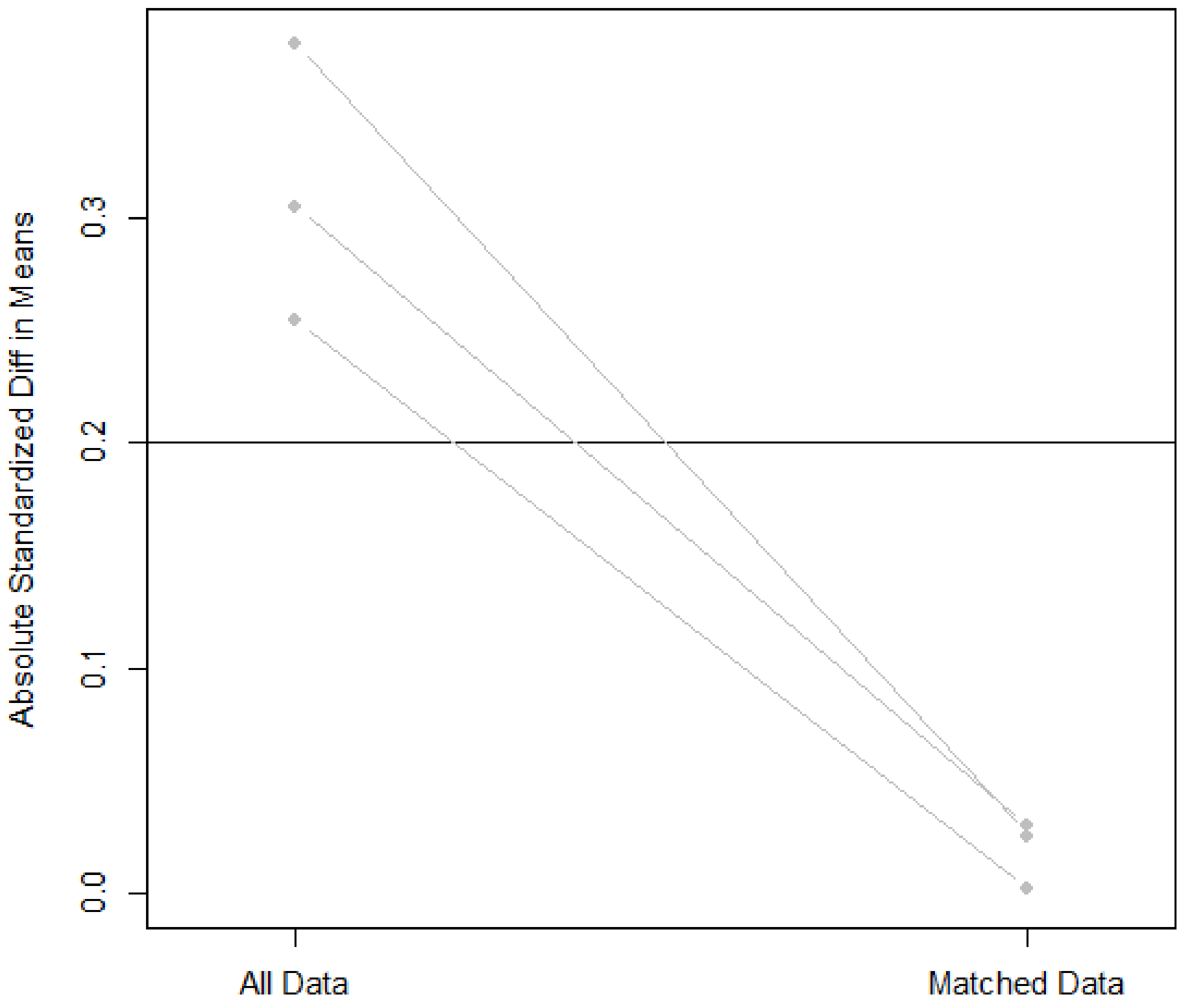
Absolute Standardized Diff in Means
0.0
0.1
0.2
0.3
All Data
Matched Data

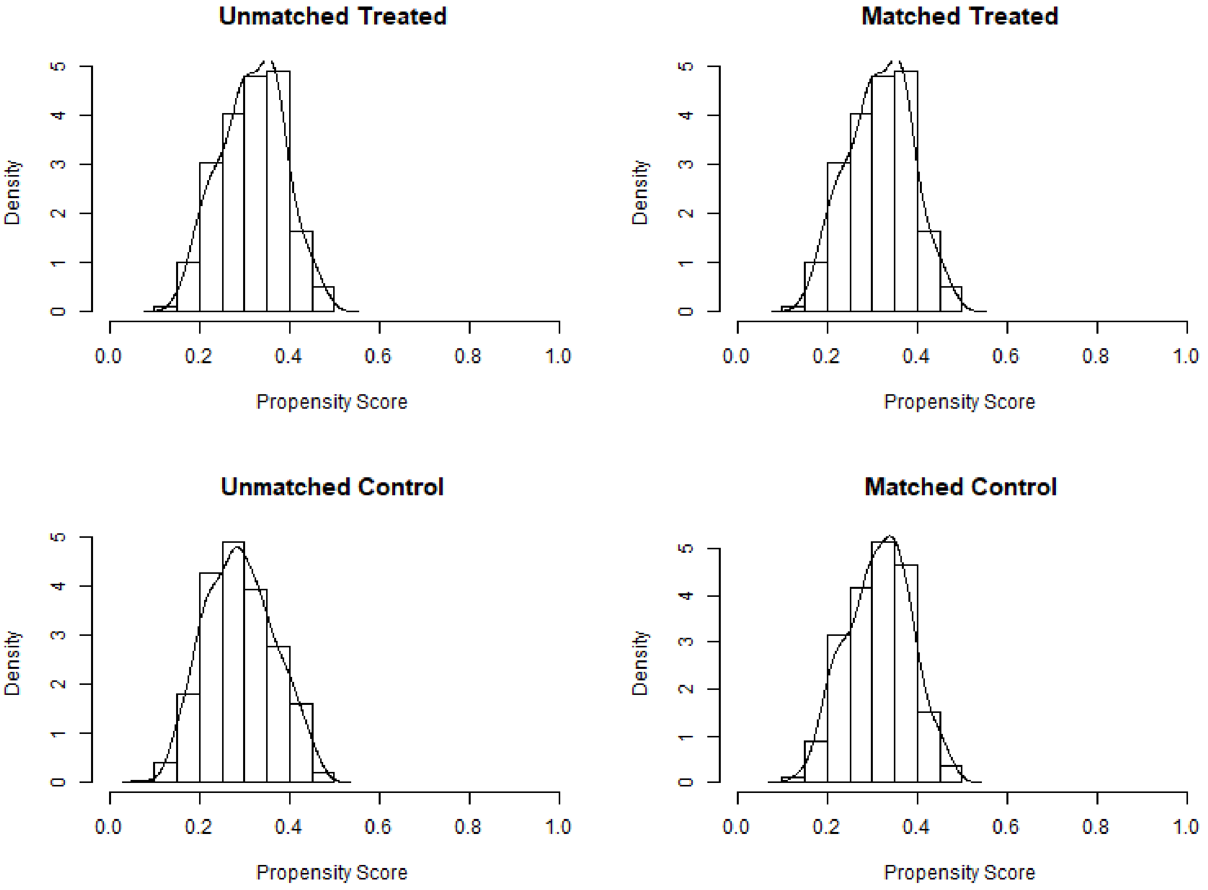
Unmatched Treated
Matched Treated
Unmatched Control
Matched Control
Density
0
1
2
3
4
5
0.0
0.2
0.4
0.6
0.8
1.0
Propensity Score

Standardized differences before matching

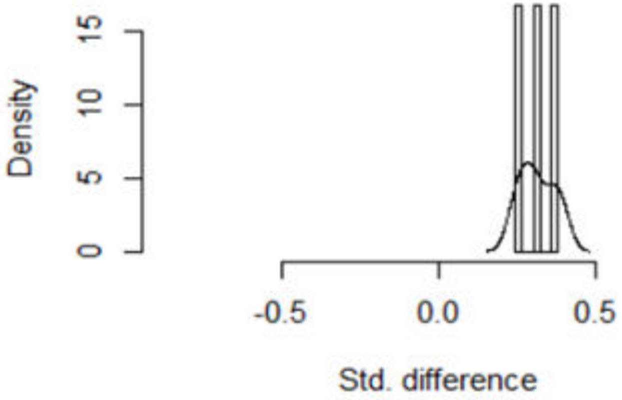

Standardized differences before matching

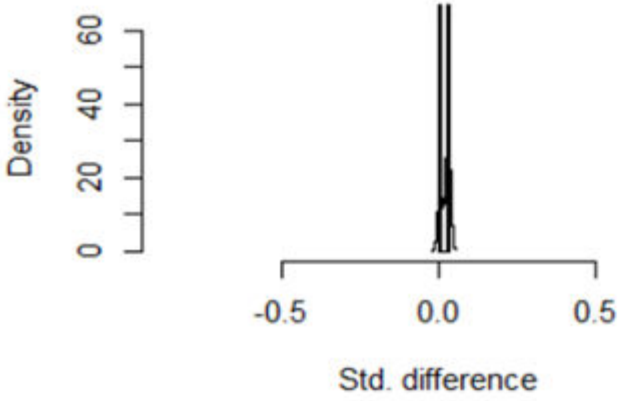

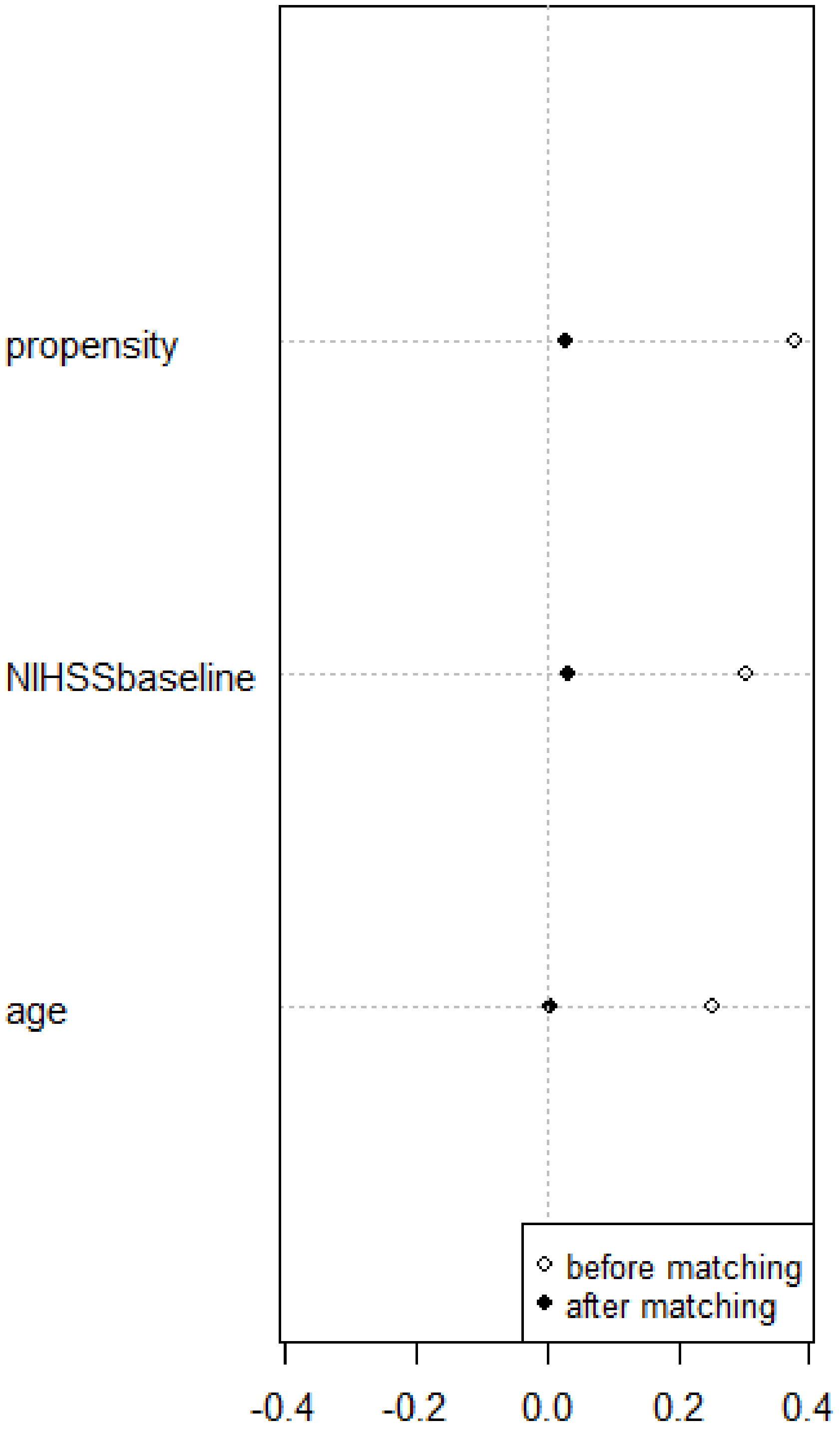
propensity
NIHSSbaseline
age
before matching
after matching
-0.4
-0.2
0.0
0.2
0.4
